# Genome-wide characterization and drought-responsive expression profiling of plasma membrane intrinsic proteins (PIPs) in *Caragana korshinskii*

**DOI:** 10.1186/s12870-026-09485-8

**Published:** 2026-07-28

**Authors:** Wenyu Li, Hatieti Madeti, Xinyi Liu, Jiahui Ren, Yuze Wang, Zhilai Wang, Peizhi Yang, Xiaoxia Shen

**Affiliations:** College of Grassland Agriculture, Northwest A&F University, Yangling, 712100 China

**Keywords:** *Caragana korshinskii*, Plasma membrane intrinsic proteins (PIPs), Genome-wide analysis, Drought stress, Expression pattern

## Abstract

Plasma membrane intrinsic proteins (PIPs) are an important subfamily of aquaporins that function as membrane channels that facilitate water transport across the plasma membrane in plants. Although the molecular mechanisms of PIPs have been extensively studied in model species, their characteristics and functions remain poorly characterized in *Caragana korshinskii*. Here, 13 *CkPIP* genes were identified through genome-wide analysis. CkPIPs were clustered into PIP1 (seven members) and PIP2 (six members) subfamilies. They exhibited conserved physicochemical properties, contained cis-acting elements responsive to abiotic stresses and phytohormones, and showed evidence of segmental duplication under purifying selection. Phylogenetic, structural, and expression analyses (RNA-seq and RT-qPCR) were performed to explore their evolutionary patterns and responses to drought stress. Under drought stress, *CkPIPs* exhibited tissue-specific expression dynamics: *CkPIP1;1*, *CkPIP1;2*, and *CkPIP2;2* were prominently upregulated, indicating that they play important roles in drought adaptation. This study expands our understanding of the characteristics of the CkPIP family and provides valuable molecular insights for drought-resistance research and forage improvement.

**Supplementary Information:**

The online version contains supplementary material available at https://doi.org/10.1186/s12870-026-09485-8.

## Introduction

*Caragana korshinskii* Kom, commonly known as the pea shrub, is a small leguminous shrub belonging to the family Fabaceae. This species exhibits remarkable traits such as tolerance to barrenness, resistance to salinity, cold hardiness, drought endurance, and high biomass yield [1]. It is characterized by high nutritional value and palatability and is widely distributed across sandy grasslands in the arid and semi-arid regions of northwestern China and Mongolia. Long-term adaptation to drought and saline environments has driven the evolution of specialized physiological mechanisms conferring exceptional drought resistance [2]. Its abundant flowering also makes it an important nectar-producing species. *C. korshinskii* plays a vital ecological role as a key species in windbreak, sand-fixation, and soil–water conservation forests. Its cultivation contributes to the restoration of degraded pastures, enhances the use of saline soils, and supports food security, sustainable animal husbandry, and ecological conservation [3].

Drought is among the most severe abiotic stresses limiting plant growth and productivity worldwide. To cope with water scarcity, plants have evolved diverse adaptive strategies at the morphological, physiological, and molecular levels [4]. Among these mechanisms, maintaining water transport and cellular homeostasis is crucial for sustaining drought tolerance. Plasma membrane intrinsic proteins (PIPs), a subfamily of aquaporins, play an essential role in facilitating water transport across cell membranes [5]. PIPs help regulate water balance, stomatal movements, and osmotic responses. As key components in plant drought adaptation, PIPs can indirectly influence plant growth, biomass accumulation, and tissue tenderness. These traits are important determinants of forage quality and palatability [6]. These proteins are small intrinsic membrane proteins characterized by major intrinsic protein (MIP) domains and localized to the plasma membrane [7]. Their primary function involves forming selective pores that enable the regulated, single-file movement of water molecules across the membrane. In arid and semi-arid environments, improved water-use efficiency and maintenance of cellular hydration are closely associated with enhanced biomass production and plant performance. Therefore, elucidating the regulatory roles of *PIP* genes in drought-adapted species such as *C. korshinskii* may provide a molecular basis for improving not only stress tolerance but also forage performance [8].

Plant PIPs are classified into two subfamilies, PIP1 and PIP2, based on their sequence similarity [5, 9]. Phylogenetic analyses have revealed the presence of PIPs in both bryophytes and gymnosperms, indicating that the divergence between the PIP1 and PIP2 subfamilies has likely occurred before the evolutionary split between monocots and dicots [10]. Structurally, PIP1 and PIP2 differ primarily in the lengths of their N- and C-terminal regions, which correspond to distinct functional properties [11]. PIP2 proteins are highly efficient in that facilitate water transport across the plasma membrane, whereas PIP1 proteins show lower water permeability and are often associated with the transport of small solutes. PIP1 proteins typically require co-expression with PIP2 proteins for proper membrane localization, highlighting their cooperative roles in regulating cellular water and solute dynamics [12]. Moreover, enhanced expression of PIP genes in transgenic plants has been linked to increased stomatal conductance, leaf expansion, and organ development [13].

Despite the recognized importance of PIPs in drought responses, most studies have focused on model organisms such as *Arabidopsis thaliana* and *Oryza sativa*. In *A. thaliana*, PIP1 and PIP2 proteins are actively involved in responses to abiotic stresses, including drought and salinity [14]. Empirical evidence indicates that the expression of these proteins decreases under salt stress, leading to delayed recovery of hydraulic conductance and transpiration rates. Conversely, overexpression of *OsPIP1-1* or *OsPIP2-2* enhances tolerance to both salt and drought stress [15]. However, extremophytic species such as *C. korshinskii*, known for their remarkable drought tolerance, have received limited attention. Investigating the role of PIPs in such species could provide novel insights into the molecular mechanisms underlying drought adaptation. Furthermore, the distinct evolutionary history and environmental adaptations of *C. korshinskii* make it an excellent model for exploring drought tolerance mechanisms in non-model plants.

A substantial gap remains in our understanding of the PIP gene family in *C. korshinskii.* Although PIP genes have been extensively characterized in model plants, their diversity, evolutionary relationships, structural features, and potential regulatory roles in forage species remain poorly understood. Moreover, the expression patterns of PIP genes under drought stress and the potential contributions of PIP genes to drought tolerance in *C. korshinskii* have not been systematically investigated. Addressing these gaps is essential for advancing our understanding of plant drought adaptation mechanisms and for identifying candidate genes that may contribute to drought tolerance in forage plants adapted to arid and semi-arid environments.

In this study, we have performed a comprehensive genome-wide analysis of the PIP gene family in *C. korshinskii.* Our aim was to clarify their evolutionary relationships, gene structures, and conserved features, to characterize their genomic distribution and cis-regulatory elements, and to explore their potential protein interaction networks. We further sought to investigate the transcriptional responses of *CkPIP* genes to drought stress at different tissues and time points, with the aim of identifying key family members potentially mediating drought adaptation. To achieve this goal, we integrated phylogenetic analysis, gene structure and promoter motif characterization, protein–protein interaction prediction, and transcriptome-based expression profiling, which was further validated via RT-qPCR assays. Collectively, these results provide insights into the molecular framework underlying aquaporin-mediated drought tolerance in *C. korshinskii* Our findings further establish a foundation for subsequent functional gene studies, the enhancement of drought resilience in forage plants, and ecological restoration initiatives in arid regions.

## Materials and methods

### Plant material

Seeds of *C. korshinskii* were collected in August 2022 from Chaheze Township, Yulin City, Shaanxi Province, China (altitude: 1110.94 m; GPS coordinates: 109° 59′ 42″ E, 38° 50′ 32″ N). The collected seeds were preserved at the College of Grassland Science, Northwest A&F University. Genomic sequencing of *C. korshinskii* was previously performed. No voucher specimens were prepared for this study, as the plant material was sampled from a well-documented, widely recognized natural population. Species identification was conducted via established morphological traits and local floristic record archives. Seeds were sterilized with 75% ethanol three times for 3 min, with subsequent rinsing using distilled water, and then incised with a sterile scalpel. Seeds were placed on moistened filter paper (24 h, dark incubation at 28 °C), stratified at 4 °C for 3 days. Following stratification, germinated seedlings were transferred to a growth chamber (16 h light/8 h dark photoperiod, 22 ± 1 °C) and cultivated in a substrate mixture of nutrient soil, vermiculite, perlite (1:1:3). Drought stress treatment was initiated when seedlings reached 60 days old (approximately two months after sowing), at which point they attained a well-established vegetative stage. Leaf samples were collected at 0, 3, 6, 12, and 24 days post-stress (3 seedlings per replicate, 3 biological replicates), immediately frozen in liquid nitrogen, and stored at − 80 °C for subsequent RNA extraction and RT-qPCR analysis.

### Genome-wide identification of the PIP gene family in C. korshinskii

To comprehensively identify putative *CkPIP* genes in *C. korshinskii*, we used the published genome assembly and annotation dataset of *C. korshinskii* [11]. The hidden Markov model (HMM) profile of the conserved major intrinsic protein (MIP) domain (PF00230) was downloaded from the Pfam database and used to search against the *C. korshinskii* genome database using HMMER v3.0 integrated within TBtools v2.420. All candidate proteins were further validated using the NCBI Conserved Domain Database (CDD). Sequences that lacked the complete MIP domain or were identified as redundant were manually removed to retain high-confidence CkPIP members. Physicochemical properties, including amino acid sequence length, molecular weight, isoelectric point, instability index, aliphatic index, and grand average of hydropathicity (GRAVY) value, were predicted using two online tools: ExPASy (http://web.expasy.org/compute_pi) and Plant-mPLoc (http://www.csbio.sjtu.edu.cn/bioinf/plantmulti/)..

### Multiple sequence alignment, phylogenetic analysis, and tertiary structure prediction

We performed phylogenetic analysis using the full-length amino acid sequences of all CkPIP proteins from *C. korshinskii* together with representative aquaporins from several plant species, including *Arabidopsis thaliana*, *Glycine max*, *Medicago truncatula*, and *Pisum sativum*. Multiple sequence alignment was conducted using ClustalW, and we constructed the phylogenetic tree via the Neighbor-Joining (NJ) method with 1000 bootstrap replicates in MEGA 11. The resulting tree was visualized and annotated using Evolview v3 (https://www.evolgenius.info/evolview/). Conserved MIP domains in all CkPIP proteins were aligned and visualized using ggMSA with default parameters. The tertiary structure of a representative CkPIP protein was independently predicted using the AlphaFold Database (https://alphafold.ebi.ac.uk/).

### Gene structures, conserved motifs, and cis-acting element analysis

Conserved motifs within CkPIP proteins were identified using MEME Suite 5.4.1 (http://meme-suite.org/tools/meme) with default parameters, except for the maximum number of identified motifs, which was manually set. Cis-acting regulatory elements were predicted within the 2-kb promoter regions of each of the *CkPIP* genes using PlantCARE (http://bioinformatics.psb.ugent.be/webtools/plantcare/).

### Chromosome localizations and CkPIP genes synteny analysis

Chromosomal locations of *CkPIP* genes were mapped using MapInspect (http://www.softsea.com/download/MapInspect.html), based on the *C. korshinskii* genome annotation (GFF3 format). The resulting chromosomal positions were further visualized using TBtools with Circos visualization mode.

### Protein–protein interaction network analysis

A protein–protein interaction (PPI) network of the CkPIP proteins was predicted using the STRING database (https://string-db.org). Since *C. korshinskii* is not currently annotated in the STRING database, homologous proteins from *G. max* (a closely related Fabaceae species with well-curated PPI datasets) were adopted as reference sequences. The predicted interaction network was visualized using Cytoscape v3.9.1 [16].

### Heatmap analysis and RT-qPCR verification of the transcriptome data

Differentially expressed *CkPIP* genes were identified from RNA-seq data under the thresholds of FDR ≤ 0.05 and |log₂FC|≥ 1. RNA-seq samples were collected at 0, 7, and 14 days of drought treatment. Libraries were constructed and sequenced on the Illumina sequencing platform. The raw RNA-seq datasets have been deposited in the National Genomics Data Center (NGDC, China National Center for Bioinformation) under BioProject accession number PRJCA030077. Clean reads were mapped to the published *C. korshinskii* reference genome, and gene expression levels were calculated as FPKM values. Heatmaps were used based on log₂-transformed FPKM values using TBtools.

Subsequently, RT-qPCR assays were performed using FastReal qPCR PreMix (SYBR Green; Tiangen, Beijing, China) on a Roche LightCycler 480 system. Samples were collected at 0, 3, 6, 12, and 24 days post-stress to achieve higher temporal resolution for expression validation. Primer sequences were listed in Supplementary Table S1. Relative expression levels were calculated via the 2^−ΔΔCt^ method, with *CiEF1α* (GenBank No.: KC679842) selected as the internal reference gene [17, 18]. Each assay contained three independent technical replicates.

## Results

### Systematic identification of the CkPIP gene family

Using a hidden Markov model (HMM) to characterize the conserved MIP domain, we identified 13 PIP family members in *C. korshinskii*. Comprehensive analysis of these CkPIP proteins revealed considerable diversity in their physicochemical properties, providing insights into their potential biological significance. Amino acid lengths ranged from 187 residues in CkPIP2;5 to 289 residues in CkPIP1;1 and CkPIP1;2 (Supplementary Table S2). Such sequence variation may affect protein conformation and function; longer sequences may facilitate complex folding and intermolecular interactions, whereas shorter sequences such as CkPIP2;5 may confer greater structural flexibility and higher turnover rates.

The molecular weights of CkPIP proteins ranged from 19.90 kDa (CkPIP2;5) to 31.13 kDa (CkPIP1;5), which were determined by their amino acid composition and sequence length. Isoelectric points (pI) ranged from 6.58 (CkPIP2;3) to 9.92 (CkPIP1;5 and CkPIP1;6), indicating distinct biochemical characteristics under physiological conditions. Proteins with higher pI values tend to carry positive charges at physiological pH, favoring interactions with negatively charged molecules including membranes and nucleic acids, while those with lower pI values preferentially bind to positively charged components [19].

All CkPIP proteins exhibited instability index values below 40, as determined using the ExPASy ProtParam tool. Positive GRAVY values were observed for all CkPIP proteins, indicating overall hydrophobic protein properties. The aliphatic index ranged from 92.81 (CkPIP2;3) to 111.31 (CkPIP1;6). Detailed physicochemical parameters, including molecular weight, isoelectric point, instability index, GRAVY, and aliphatic index, were summarized in Supplementary Table S2.

### Phylogenetic and classification analysis of CkPIP proteins

**Fig. 1:**
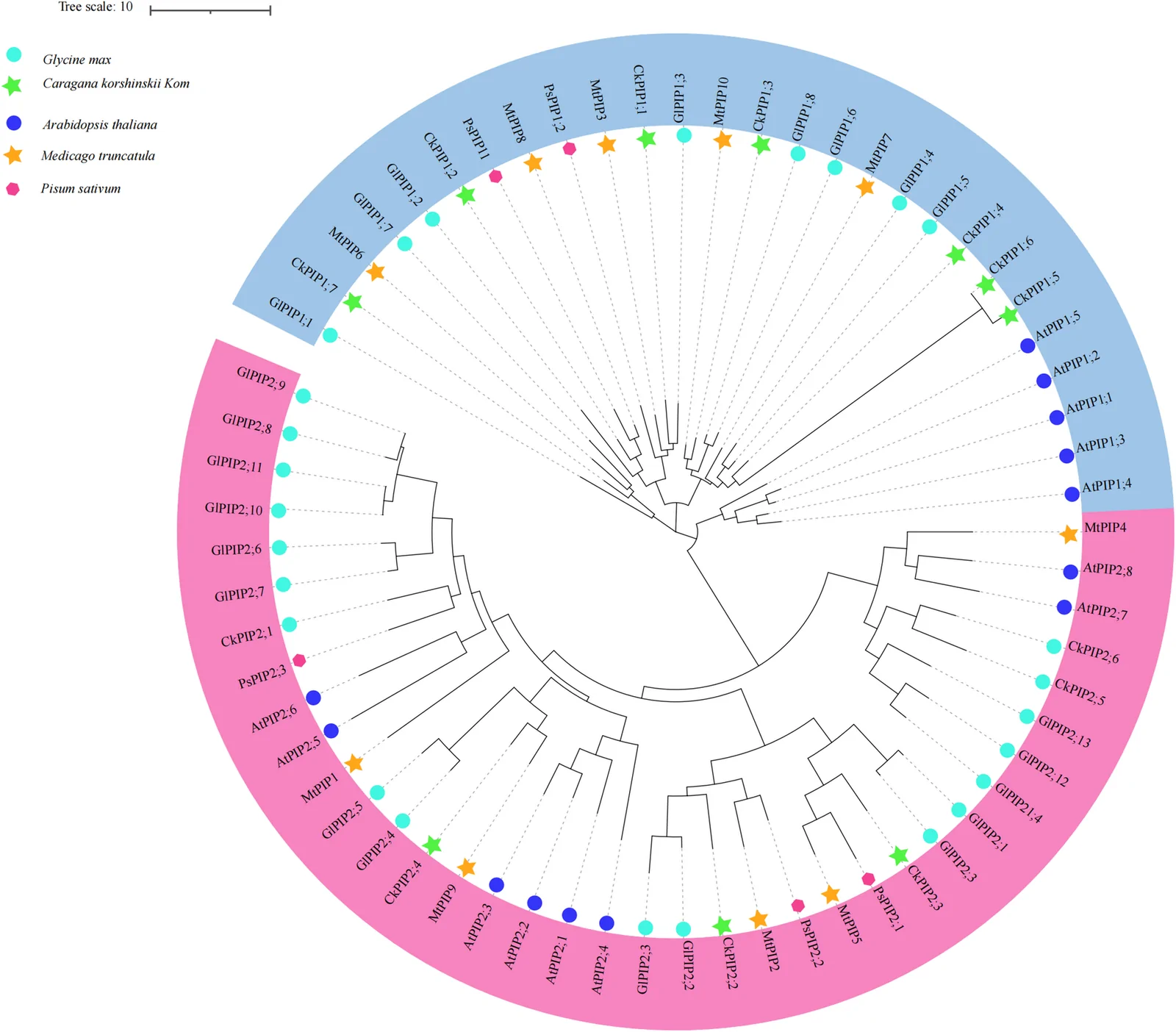
Phylogenetic tree of PIP proteins from *Caragana korshinskii*, *Arabidopsis thaliana*, *Glycine max*, *Medicago truncatula*, and *Pisum sativum*. The tree was constructed based on full-length amino acid sequences using the Neighbor-Joining method with 1000 bootstrap replicates. Different symbols represent PIP proteins from different species. The PIP family is divided into two subgroups, PIP1 and PIP2

To investigate the phylogenetic relationships of PIP proteins, we constructed a phylogenetic tree using full-length amino acid sequences from *Caragana korshinskii*, *Arabidopsis thaliana*, *Glycine max*, *Medicago truncatula*, and *Pisum sativum* (Fig. 1). The results revealed clear separation of all PIP proteins into two distinct subgroups, PIP1 and PIP2. CkPIP proteins were present in both subgroups and distributed across multiple branches alongside PIP proteins from the other four species. Notably, CkPIP proteins clustered closely with aquaporins from legume species, including *G. max*, *M. truncatula*, and *P. sativum*, indicating a relatively conserved evolutionary relationship within the legume family.

### Conserved motifs and gene structure of the CkPIP gene family

**Fig. 2:**
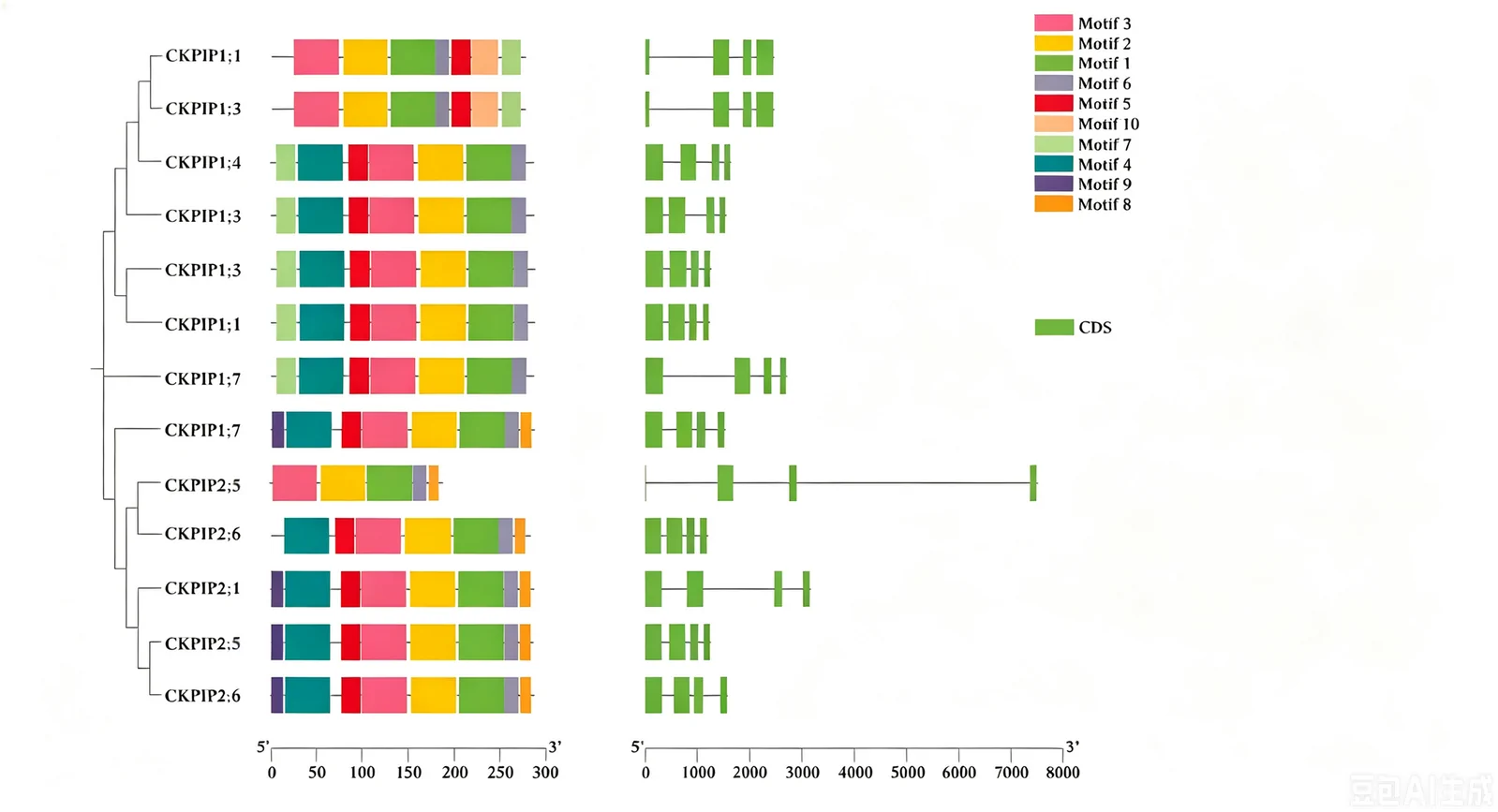
Phylogenetic relationship, conserved motifs, and gene structure analysis of *CkPIP* family members of the *C. korshinskii* genome

To investigate the sequence characteristics of the *CkPIP* gene family, we performed systematic analyses of conserved motifs and gene structure. A total of ten conserved motifs were identified across the 13 CkPIP members using the MEME Suite. Members belonging to the same subfamily exhibited strong conservation in both motif type and number. Among these, Motifs 1, 2, 3, 4, 5, and 7 were frequently observed, while Motifs 3, 4, and 5 were present in all family members, highlighting their critical role in maintaining PIP protein structural and functional integrity (Fig. 2).

Subfamily-specific variations were clearly evident. For example, Motif 8 was exclusively present in all PIP2 members but completely absent from PIP1 members, suggesting specialized functions associated with PIP2 subfamily. Gene structure analysis further revealed distinct evolutionary patterns within the family. Members within the same subfamily shared highly similar exon–intron organizations. Most PIP1 members exhibited consistent exon numbers and intron positions, aligning well with their conserved motif profiles, consistent with functional redundancy and evolutionary conservation. In contrast, inter-subfamily variation was observed, as some PIP2 members displayed divergent intron lengths or distinct exon–splicing patterns, which correlated with their unique subfamily-specific motifs such as Motif 8.

The interplay between gene structure and motif composition likely reflects the evolutionary history of the *CkPIP* gene family. Our results suggest that conserved motifs within stable gene structures serve to maintain essential protein functions. Conversely, subfamily-specific structural features and associated motifs may further contribute to functional divergence within the gene family (Fig. 2).

### Three-Dimensional modeling prediction and network analysis of CkPIP proteins

**Fig. 3:**
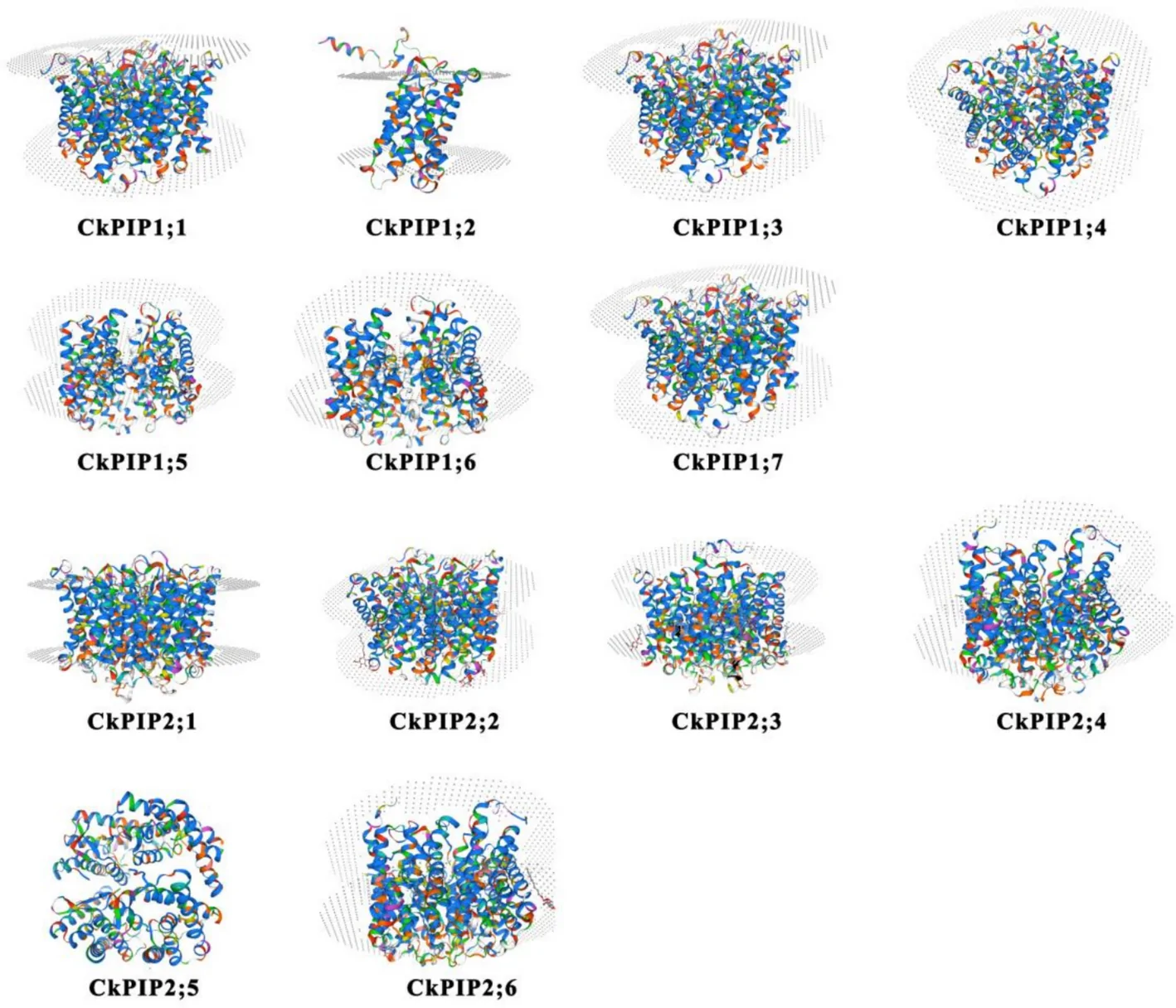
Predicted tertiary structures of the selected 13 CkPIP protein using the AlphaFold 3. Modeled at a 100% confidence level by using Phyre2 server and the percentage residues ranged from 78 to 98%

To investigate the structural characteristics of the *C. korshinskii* PIP family and their potential associations with abiotic stress responses, we predicted three-dimensional (3D) structural models for all 13 CkPIP proteins (Fig. 3), verifying high reliability of the predicted protein structures. All CkPIP proteins displayed a highly conserved hourglass-like core structure, which is presumed to be essential for canonical protein function and mediates transmembrane transport processes linked to abiotic stress tolerance.

Distinct variations in secondary structure components were detected among different CkPIP members (Supplementary Table S3). In detail, α-helix proportions ranged from 30.66% (CkPIP1;7) to 36.73% (CkPIP1;5), β-turns from 1.05% (CkPIP1;7) to 3.64% (CkPIP1;6), random coils from 43.27% (CkPIP1;6) to 52.60% (CkPIP1;1), and extended strands from 14.88% (CkPIP1;1) to 20.86% (CkPIP2;5). Such differences in secondary structure profiles may modulate protein folding efficiency, structural stability, and functional performances including substrate binding, within the conserved 3D protein architecture.

### Promoter cis-acting elements of the CkPIP gene family

**Fig. 4:**
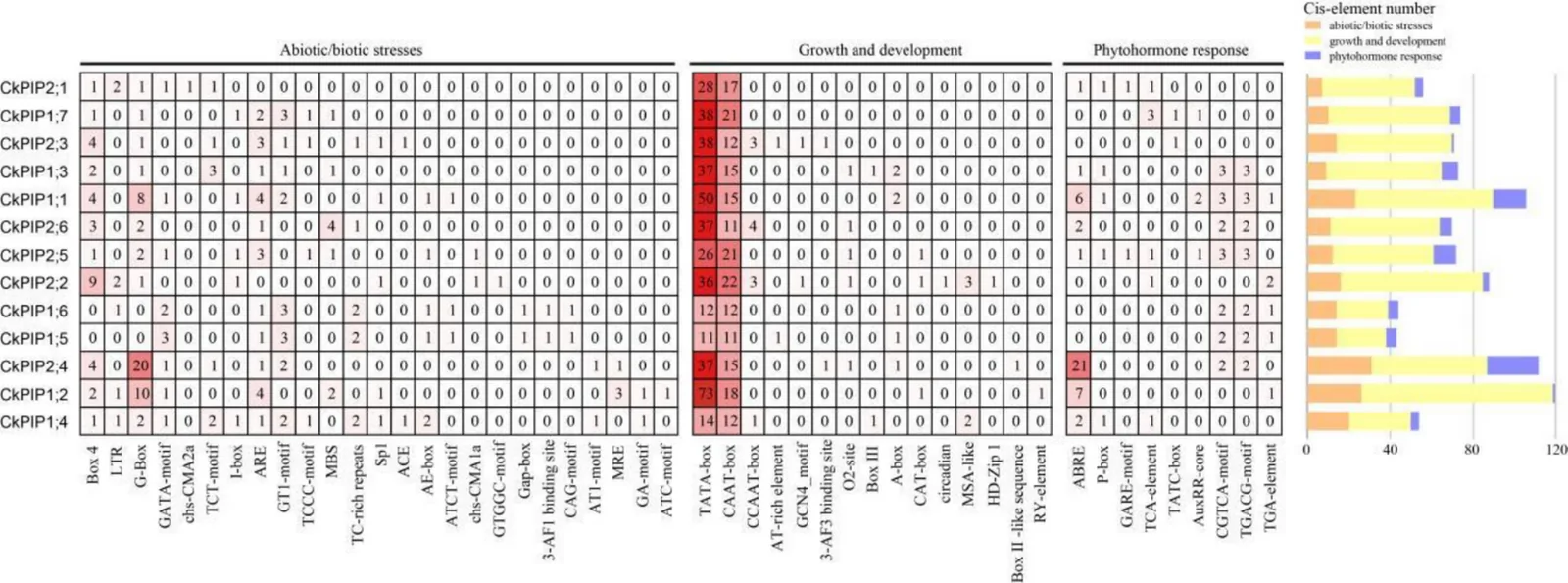
Analysis of cis-acting elements of *CkPIP* members. Three groups representing abiotic/biotic stresses, growth and development, and phytohormone response, respectively. Names of cis-acting elements are shown below

To explore the regulatory mechanisms of the *CKPIP* gene family, we systematically analyzed cis-acting elements within the 2-kb promoter regions of all 13 CkPIP members. Multiple categories of cis-elements linked to abiotic and biotic stress responses, plant growth and development, as well as phytohormone signaling pathways were identified. Stress-responsive elements primarily comprised LTR (low-temperature response), ARE (anaerobic response), and MBS (drought-inducible MYB binding site). Specifically, CkPIP1;1 harbored eight LTR elements associated with low-temperature adaptation, whereas CkPIP2;4 contained 20 ARE elements related to anaerobic stress. Growth-regulatory elements included the CAT-box (meristem expression) and circadian rhythm elements (rhythm regulation), among which CkPIP1;2 possessed 73 growth-associated elements, indicating its potential function in meristem development regulation. Moreover, abundant phytohormone-responsive elements were detected, including the TCA-element (salicylic acid response) and GARE-motif (gibberellin response), with CkPIP2;4 containing 21 hormone-related elements (Fig. 4). The heterogeneous distribution of cis-acting elements across CkPIP members underscored their extensive potential functions in stress resilience, plant growth modulation, and phytohormone signal transduction.

### Analysis of the chromosomal localization of CkPIP gene family

**Fig. 5:**
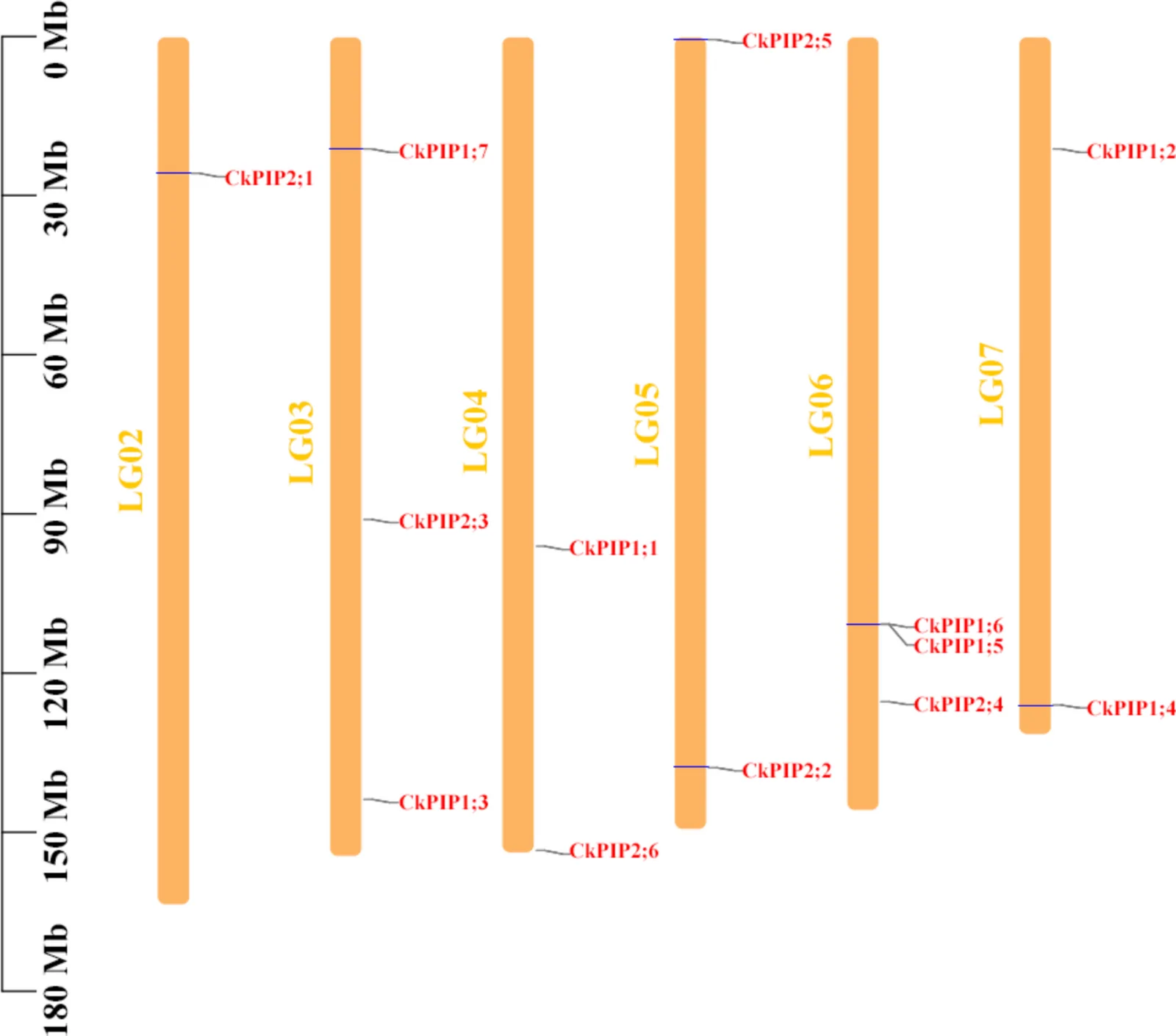
Chromosomal distributions of *CkPIP* members in the *C. korshinskii* genome

Urther analyzed the distribution of 13 *CkPIP* genes across chromosome-level linkage groups (LGs) (Fig. 5). The *CkPIP* genes were unevenly distributed across six linkage groups (LG02–LG07), whereas no *CkPIP* gene members were detected on LG01, suggesting possible reduced gene retention or evolutionary deletion. LG03 and LG04 each contained more than three *CkPIP* genes, serving as major genomic loci for this gene family. This distribution pattern may have resulted from segmental or tandem duplication events, which contributed to functional diversification within the *CkPIP* gene family. LG04, LG05, and LG07 each harbored two *CkPIP* genes, while LG02 contained only one, reflecting variable evolutionary pressures shaping gene density across different linkage groups. The absence of *CkPIP* genes on LG01 may indicate that this genomic region has limited relevance to water transport or stress adaptation, or is susceptible to genomic structural rearrangements (Fig. 5).

### Gene duplication events and collinearity analysis of the CkPIP gene family

**Fig. 6:**
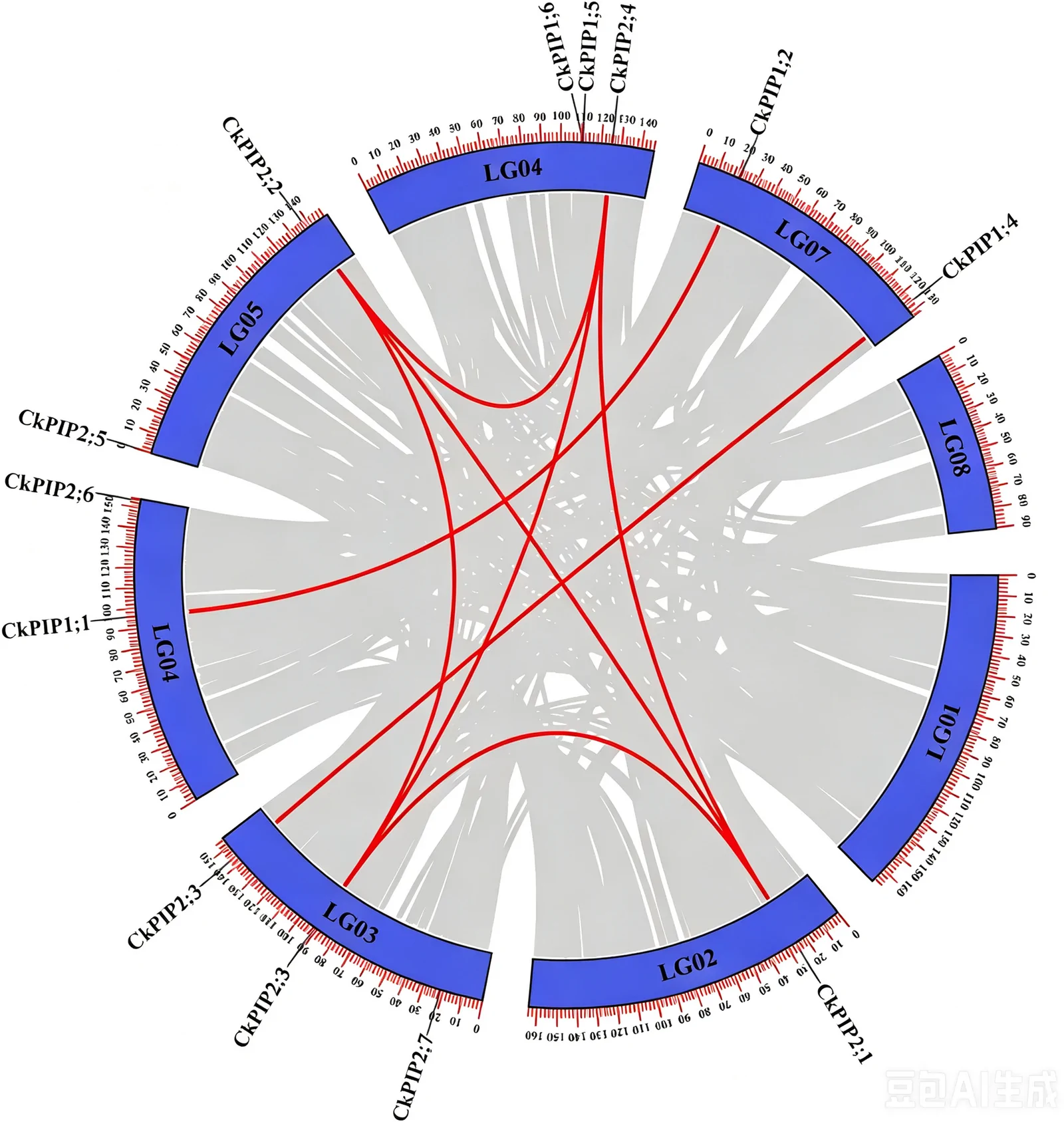
Collinearity analysis of the *CkPIP* family members. The red lines represent gene pairs with collinearity relationships, the labels in the boxes denote chromosomes, and the scale ranging from 0 to 140 on the chromosomes indicates gene density

To explore the evolutionary trajectory of the *CKPIP* gene family, we analyzed genome-wide duplication events using MCScanX, with a focus on the role of segmental duplication in gene family expansion. Eight segmentally duplicated gene pairs were identified, which formed collinear blocks across different chromosomes. Notably, *CkPIP2;2* (LG05) and *CkPIP2;4* (LG06) exhibited high sequence similarity and synteny, indicating their origin from ancient segmental duplication events that contributed to genetic diversity (Fig. 6). These duplicated gene pairs may have facilitated functional diversification, promoting specialization in water transport and stress response processes.

**Fig. 7:**
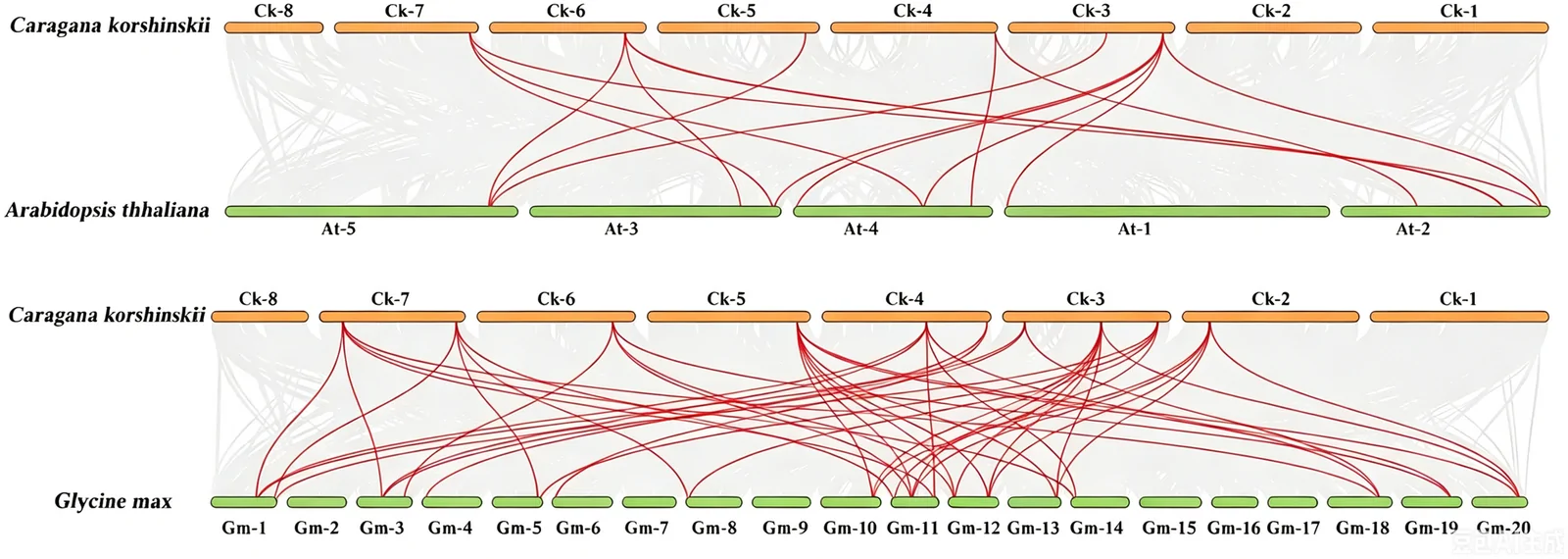
Comparative genomics analysis of *PIP* gene collinearity between *C. korshinskii* and two model plant species (*A. thaliana* and *G. max*). The gray background lines indicate blocks of collinearity within *C. korshinskii* and the other designated plant species, while the red lines denote orthologous PIP genes

Cross-species collinearity analysis between *C. korshinskii* and *A. thaliana* as well as *G. max* identified 15 and 44 collinear pairs, respectively, indicating conserved synteny across dicot species and shared evolutionary origins. These results highlighted the role of segmental duplication in *CkPIP* family expansion and the conservation of collinear blocks across distantly related species, providing a framework for understanding functional divergence and adaptive evolution of the *CkPIP* gene family (Fig. 7).

### Analysis of the protein–protein interaction network of CkPIP gene family

**Fig. 8:**
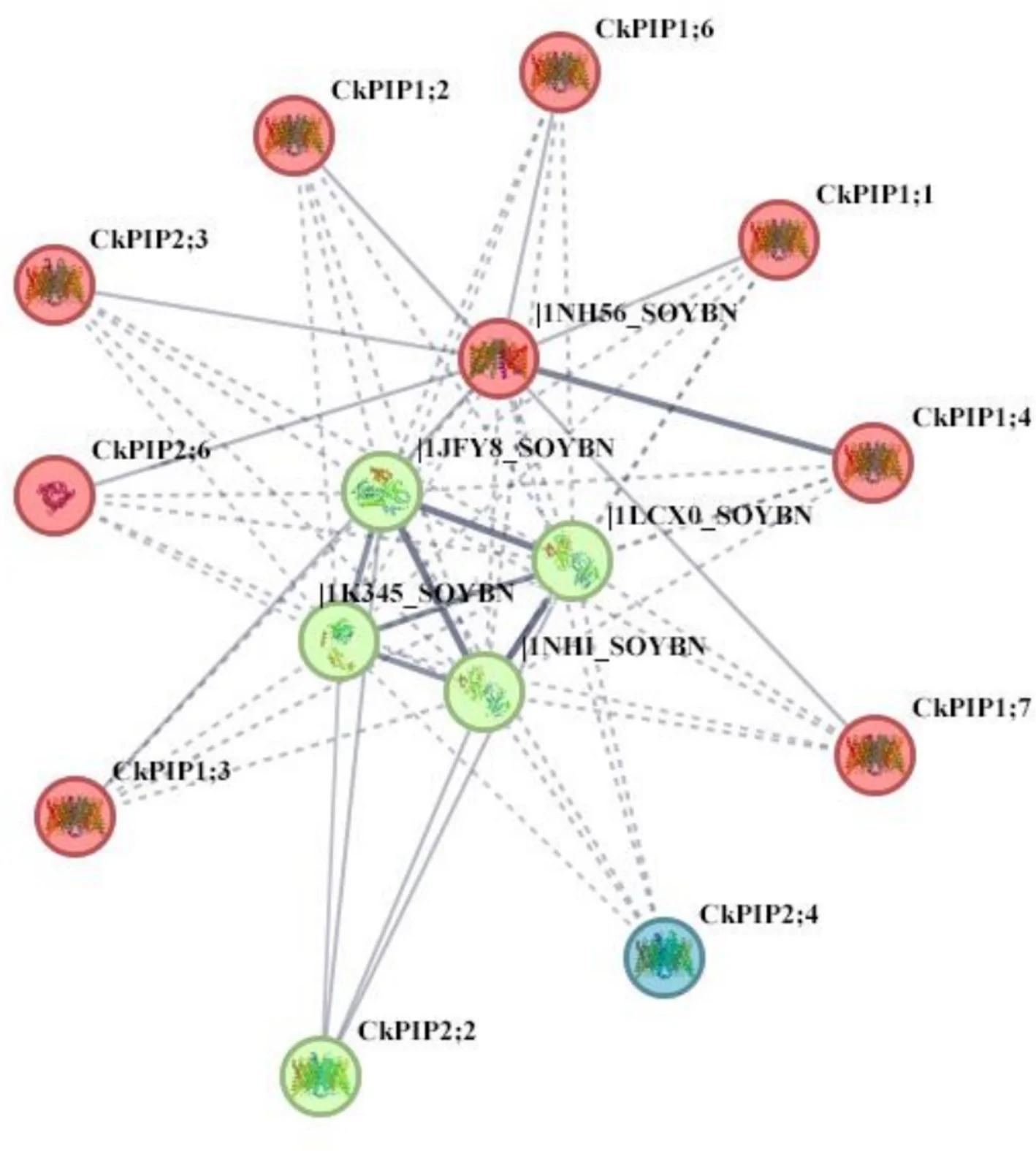
Predicted protein–protein interaction network of CkPIP proteins. Red nodes represent CkPIP1 subfamily members, blue nodes represent CkPIP2 subfamily members, and green nodes indicate predicted interacting proteins derived from soybean orthologs. Edges represent predicted protein–protein interactions, with solid lines indicating higher-confidence interactions and dashed lines indicating lower-confidence interactions. Node size is uniform and used for visualization purposes only

To further investigate the functional characteristics of the CkPIP-protein, we constructed a protein–protein interaction (PPI) network using the STRING database, with *Glycine max* protein sequences serving as a reference for comparative analysis. All 13 CkPIP proteins were integrated into the constructed network. The generated network contained 15 nodes and 59 edges, corresponding to interacting proteins and their interactive relationships. Among all CkPIP members, 10 were embedded within the predicted interaction framework, establishing interaction links with five homologous proteins from soybean. By contrast, the remaining three CkPIP proteins failed to form detectable interactions under the preset confidence threshold (Fig. 8).

Notably, network topology analysis revealed that GLYMA_20G170400 possessed the largest number of interacting partners among all identified nodes, implying its crucial regulatory role in mediating core interaction relationships associated with CkPIP proteins. In plant interaction networks, proteins with high connectivity are generally regarded as potential core regulatory hubs, whereas functional validation cannot be solely determined by topological connectivity metrics. Consistent with our results, previous network-based transcriptomic and interaction studies have screened highly connected hub genes implicated in stress adaptation and nutrient regulation in soybean [20]. Accordingly, GLYMA_20G170400 was identified as a vital candidate for subsequent functional validation. Its extensive interactive connections reflected a central hub function, which potentially modulates PIP-mediated biological processes, including transmembrane water transport, osmotic homeostasis regulation, and abiotic stress response pathways (Supplementary Table S4).

### Drought-induced spatial and temporal expression patterns of CkPIP genes

**Fig. 9:**
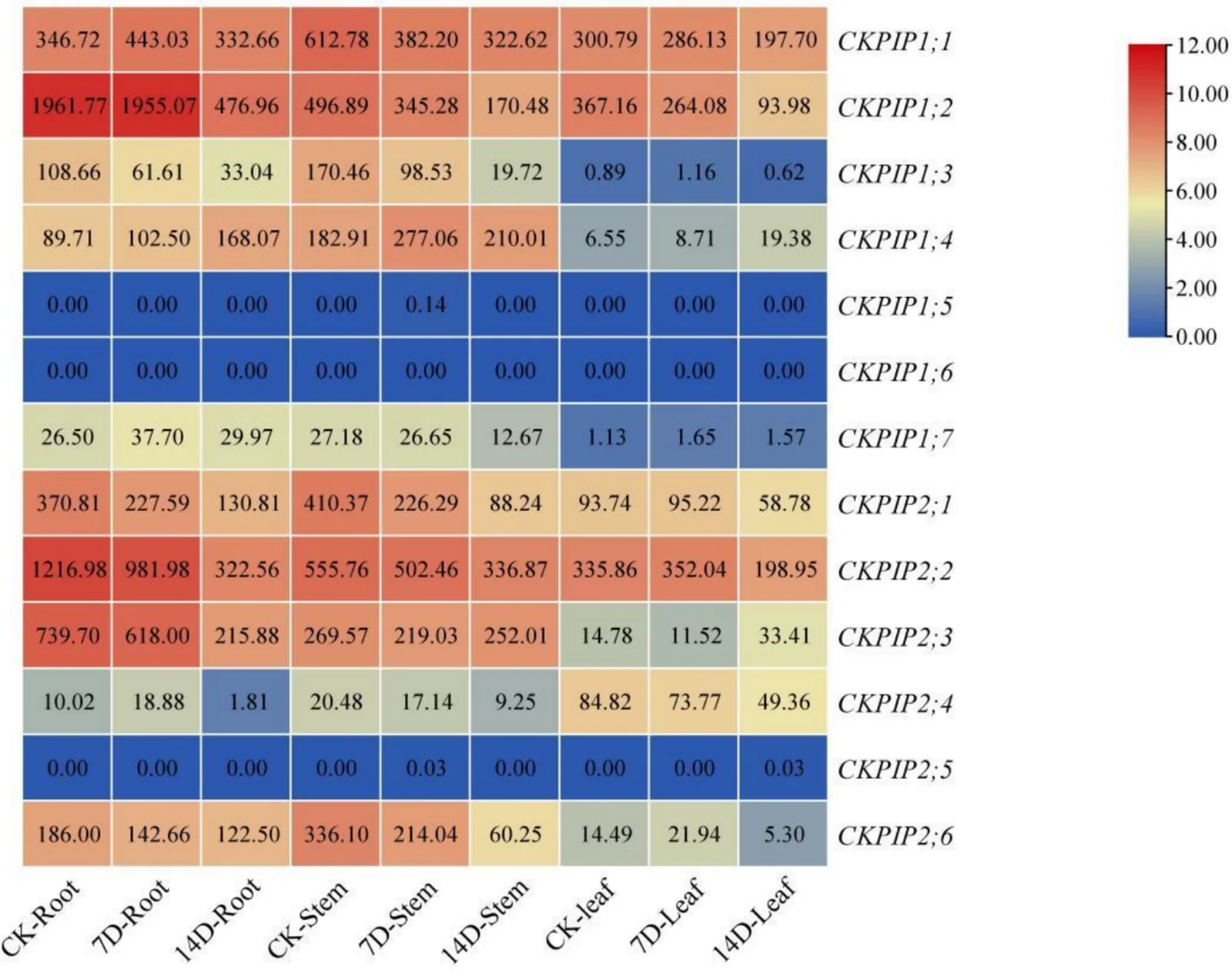
The relative expression of *CkPIP* genes in roots, stems, and leaves following drought treatments at 0, 7, and 14 days from the RNA-seq analysis. The heatmap was used based on triplicate FPKM values transformed via log₂ transformation

To analyze drought-induced expression patterns of *CkPIP* genes, we conducted RNA-seq and RT-qPCR assays under drought treatment. RNA-seq analysis was performed using root, stem, and leaf samples collected at 0, 7, and 14 days of drought treatment (Fig. 9). Transcript levels of all 13 *CkPIP* genes were detected in the three tested tissues. In stems, *CkPIP1;4*, *CkPIP2;1*, and *CkPIP2;2* were significantly upregulated at 7 and 14 days compared with the day 0 control. *CkPIP1;1*, *CkPIP1;2*, and *CkPIP2;2* exhibited higher transcript abundance across multiple tissues under drought stress. In roots, *CkPIP1;1* and *CkPIP1;2* maintained moderate to high expression levels at all sampling time points. In leaves, *CkPIP2;2* showed higher expression levels than other *CkPIP* family members.

**Fig. 10:**
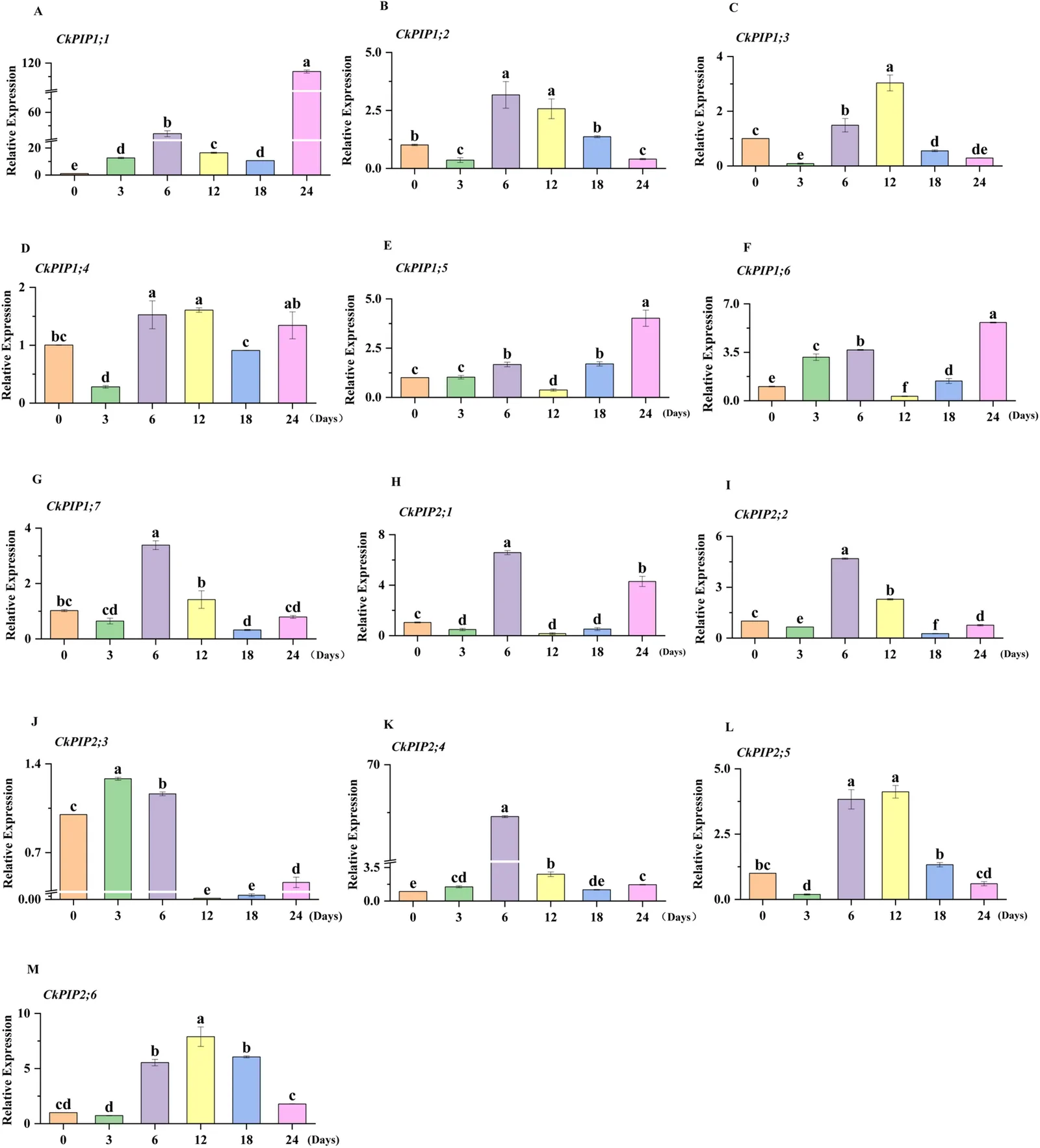
The expression levels of *CkPIP* genes in response to drought stress at 0, 3, 6, 12, and 24 days

RT-qPCR analysis was performed at 0, 3, 6, 12, and 24 days of drought treatment (Fig. 10). All 13 *CkPIP* genes displayed dynamic changes in expression during drought treatment. In leaves, transcript levels of most *CkPIP* genes increased at day 6. Specifically, *CkPIP1;2*, *CkPIP1;7*, and *CkPIP2;2* reached their maximum expression at day 6, with 3-, 2.3-, and fivefold increases relative to day 0, respectively, followed by a decrease in expression at day 24. *CkPIP1;3*, *CkPIP2;5*, and *CkPIP2;6* achieved peak expression at day 12 and then decreased at day 24. *CkPIP1;1*, *CkPIP1;5*, and *CkPIP1;6* exhibited maximum expression at day 24. Hierarchical cluster analysis grouped *CkPIP* genes with similar temporal expression patterns under drought treatment.

## Discussion

*C. korshinskii* is a perennial leguminous shrub widely distributed in arid and semi-arid regions, where long-term survival depends on efficient regulation of water balance and hydraulic adjustment. Plasma membrane intrinsic proteins (PIPs) are key components of transmembrane water transport systems and have been widely implicated in plant drought responses. Overexpression of specific PIP members in *A. thaliana*, rice, and soybean enhances drought tolerance by increasing membrane water permeability and improving hydraulic conductivity [21–24], highlighting the importance of this gene family in stress adaptation. In this context, the identification and characterization of the *CkPIP* gene family provide important insights into the molecular basis of drought adaptation in *C. korshinskii*.

Thirteen *CkPIP* genes were identified and classified into two conserved subfamilies, PIP1 and PIP2, consistent with the canonical classification reported in dicot species [5, 12]. Phylogenetic analysis revealed that several *CkPIP* proteins clustered with PIPs from leguminous species, including *Glycine max*, *Medicago truncatula*, and *Pisum sativum*, rather than with orthologs from *Arabidopsis thaliana* (Fig. 1). This pattern may reflect the evolutionary relationship among these legume species within the Fabaceae family. Similar conservation of aquaporin gene families has been reported in legumes such as soybean and *Medicago sativa* [5]. Gene structure and motif analyses further revealed strong conservation within each subfamily (Fig. 2). Members belonging to the same subfamily exhibited similar exon–intron organization and motif profiles, indicating structural stability during evolution. Motifs 3, 4, and 5 were conserved across all members, suggesting their essential roles in maintaining PIP protein structural integrity. Meanwhile, the presence of subfamily-specific motifs, such as Motif 8 present in all PIP2 members but absent from PIP1 members, indicates functional differentiation between subfamilies, consistent with structural divergence reported in *A. thaliana* and rice [25, 26].

Physicochemical analyses showed that all CkPIP proteins possessed positive GRAVY values and high aliphatic indices, reflecting strong hydrophobicity typical of membrane proteins (Supplementary Table S2). The predicted three-dimensional structures exhibited the conserved hourglass-like architecture characteristic of aquaporins, with high modeling confidence (Fig. 3). Although the overall fold was conserved, variation in secondary structure composition among members suggested potential differences in channel stability, substrate selectivity, or interaction capacity. Such structural conservation combined with structural divergence may allow CkPIPs to maintain core water transmembrane transport functions while adapting to species-specific physiological demands.

Promoter analysis revealed diverse cis-acting elements associated with abiotic stress responses and phytohormone signaling. The widespread presence of drought-responsive MBS elements, low-temperature LTR elements, and anaerobic response elements suggests that *CkPIP* transcription is tightly integrated into environmental response networks (Fig. 4). In addition, the identification of ABA-, GA-, and SA-responsive elements indicates potential regulation through hormone-mediated pathways. ABA-dependent modulation of aquaporin expression has been extensively documented under drought conditions [27, 28], and promoter activity of *MaPIP1;1* has been linked to osmotic stress regulation [28]. The differential distribution and abundance of these elements among *CkPIP* members implied distinct regulatory sensitivities, which may enable flexible and tissue-specific adjustment of hydraulic conductivity under fluctuating environmental conditions.

Chromosomal distribution and duplication analysis further illustrated the evolutionary dynamics of the *CkPIP* family (Fig. 5). The uneven distribution of genes across chromosomes and the identification of eight segmentally duplicated gene pairs suggested that genome duplication events contributed substantially to family expansion. Duplicated gene pairs with high sequence similarity may retain redundant functions or undergo subfunctionalization, allowing specialization in tissue expression or stress responsiveness (Fig. 6). Cross-species collinearity analysis revealed conserved synteny with both *A. thaliana* and *G. max*, with a greater number of collinear pairs detected with soybean (Fig. 7). This pattern reinforces the evolutionary proximity within Fabaceae and suggests that conserved genomic blocks have been maintained during divergence, while duplication-driven diversification facilitated adaptive evolution.

Protein–protein interaction prediction indicated that several CkPIPs may interact with glycerol-3-phosphate dehydrogenase (GPDH)-related proteins, including the soybean homolog GLYMA_20G170400 (Fig. 8). GPDH is involved in glycerolipid metabolism and stress responses [20, 29, 30], processes closely associated with membrane stability and osmotic adjustment. Although experimental validation is required, the predicted network suggested that CkPIPs may participate in broader regulatory systems integrating water transport with metabolic and stress-related pathways. Such integration could enhance coordination between membrane permeability, osmotic balance, and stress signaling under drought conditions.

Expression analyses provided functional clues supporting the involvement of *CkPIPs* in drought adaptation. RNA-seq data demonstrated that all members were expressed in roots, stems, and leaves, with several genes, including *CkPIP1;1*, *CkPIP1;2*, and *CkPIP2;2*, showing relatively high transcript abundance across tissues under drought treatment (Fig. 9). Similar drought-responsive expression patterns have been reported in other species [31, 32], indicating conserved roles of PIP genes in abiotic stress adaptation. RT-qPCR further revealed dynamic temporal regulation during drought progression. Some genes reached peak expression at early or intermediate stages, whereas others exhibited maximal induction at later stages, suggesting coordinated regulation during different phases of stress (Fig. 10). Early induction may contribute to rapid osmotic adjustment, while sustained or late-stage expression may support long-term maintenance of hydraulic conductivity.

*CkPIP1;1* and *CkPIP2;2* displayed similar progressive upregulation under drought treatment, with transcript levels increasing throughout the stress period. Previous studies have demonstrated that PIP1 proteins often require interaction with PIP2 members for efficient plasma membrane localization and full channel activity [33]. The parallel induction observed in this study was consistent with cooperative regulation between *PIP1* and *PIP2* subfamilies, potentially enhancing membrane water permeability under prolonged drought conditions. In roots, sustained expression of *CkPIP1;1* and *CkPIP1;2* further suggests their contribution to maintaining water uptake capacity during soil drying, which is critical for survival in arid environments.

Overall, the present study integrates evolutionary, structural, regulatory, and transcriptional evidence to characterize the *CkPIP* gene family. The conservation of core aquaporin architecture, in combination with duplication-driven diversification and drought-responsive expression patterns, supports the hypothesis that *CkPIPs* contribute to hydraulic regulation and environmental adaptation in *C. korshinskii*. Although functional validation remains necessary, the identified candidate genes, particularly *CkPIP1;1*, *CkPIP1;2*, and *CkPIP2;2*, represent promising targets for future heterologous expression and physiological analyses aimed at elucidating their precise roles in drought tolerance [13, 34–37].

## Conclusions

In this study, 13 *CkPIP* genes were identified and systematically characterized from the *C. korshinskii* genome. Phylogenetic and structural analyses revealed their classification into PIP1 and PIP2 subfamilies, with conserved features. Expression profiling demonstrated that several *CkPIP* genes, particularly *CkPIP1;1*, *CkPIP1;2,* and *CkPIP2;2*, responded to drought stress, suggesting potential roles in drought adaptation. Although these expression analyses provided insights into drought-responsive regulation, the study primarily focused on aboveground tissues. Given the central role of roots in water uptake and hydraulic regulation, future investigations that incorporate root-specific transcriptomic and physiological analyses will be essential to further clarify the functional roles of *CkPIP* genes in drought tolerance. Overall, these findings provide a foundation for future studies investigating aquaporin-mediated drought tolerance and support the potential application of *CkPIP* genes in improving stress resilience in forage species in arid environments.

## Supplementary Information

Supplementary Material 1: Supplementary Table S1 Primer information. Supplementary Table S2 The basic physicochemical properties of proteins encoded by the* CkPIP* family genes. Supplementary Table S3 CkPIP protein secondary structure information. Supplementary Table S4 Interacting protein information

## Data Availability

The genome sequence used in this study was obtained from the published genome resource of Caragana korshinskii reported by Mei et al. (2025). The RNA-seq datasets generated during the current study have been deposited in the National Genomics Data Center (NGDC, China National Center for Bioinformation，https://ngdc.cncb.ac.cn/gsub/submit/bioproject/PRJCA030077) under BioProject accession number PRJCA030077.

## Abbreviations

PIP: Plasma membrane Intrinsic Proteins
MIP: Major Intrinsic Protein
RT-qPCR: Reverse Transcription Quantitative Real-Time Polymerase Chain Reaction
FPKM: Fragments per Kilobase of Transcript per Million Mapped Reads
Ka: Nonsynonymous Substitution Rate
Ks: Synonymous Substitution Rate
TF: Transcription Factor
PPI: Protein–Protein Interaction
HMM: Hidden Markov Model
CDD: Conserved Domain Database
CiEF1α: Cyclophilin EF1α
GPDH: Glycerol-3-Phosphate Dehydrogenase
NCBI: National Center for Biotechnology Information
STRING: Search Tool for the Retrieval of Interacting Genes/Proteins
ATG: Autophagy-related
DTT: Dithiothreitol
ER: Endoplasmic reticulum
HSP: Heat shock protein
HSR: Heat shock response
UPR: Unfolded protein response
