# Supplementary material for: Genome-wide characterization and drought-responsive expression profiling of plasma membrane intrinsic proteins (PIPs) in Caragana korshinskii
Source: BMC Plant Biol. 2026 Jul 28;26:1821. doi: 10.1186/s12870-026-09485-8 (PMC13693146; doi:10.1186/s12870-026-09485-8)
Supplement: Supplementary file 1 [file 12870_2026_9485_MOESM1_ESM.docx]

**Table. S1 Primer information**

| Primer name | Forward sequence | Reverse sequence |
| --- | --- | --- |
| *CiEFα* | CAAAAAGTCCCCTCGTTGTCTC | AGCAATCGTTCTTCCTAATGATCTAA |
| *CkPIP1;1* | ACAGATCCGATTCCAAGTGCAA | TCCTCGCCAAGAACAAACCG |
| *CkPIP1;2* | AACCGTTGGTATTCAAGGAATTGC | TCAACGACAATTTCCTCGCCAAA |
| *CkPIP1;3* | CTGCACTGCTGGAATCTCA | CCAGCACCACAAATAGCTC |
| *CkPIP1;4* | ATCACGGGAACTGGCATT | CAACCCAGAAAATCCAATGGTC |
| *CkPIP1;5* | CAGATAGTGATCCGAGCAA | TGTTACGAAGACGCCAAG |
| *CkPIP1;6* | TTGGACATTTCATTGGAGC | GGTAATTCAAAACAAGCACAG |
| *CkPIP1;7* | CATTTGTTCTTGTTTATACCGTCT | TAGGAATTGTAGCCAAATGCAC |
| *CkPIP2;1* | GCTTTTCCTCTACGTTACTGTGC | AAACAAGGACGAAGATCATGCC |
| *CkPIP2;2* | AGCACCATTCTTCGACCC | CTCTGTCTGCTGTTGCCAA |
| *CkPIP2;3* | TCATCGCCGAATTCATAGCAACC | AAAATGCCAACACCATCACAGTC |
| *CkPIP2;4* | AAAGACATCGAAACCGAAGCTC | ATGAGGGCTCTGTAAAAGGACCA |
| *CkPIP2;5* | AGCCGTATGTTACATGATAGCACA | GCAGAGCCTTTGTTGTAACCAGA |
| *CkPIP2;6* | TCGCCGAGTTCATCGCCACA | GATCATGCCACCGAAGGACCAG |

**Table. S2 The basic physicochemical properties of proteins encoded by the *CkPIP* family genes**

| Gene Name | Amino acid  Length/aa | Molecular  Weight/Da | PI | Instability  index | Aliphatic  index | Grand average of hydropath-city | Subcellular  localization |
| --- | --- | --- | --- | --- | --- | --- | --- |
| *CkPIP1;1* | 289 | 31071.01 | 8.68 | 33.37 | 92.87 | 0.324 | plas |
| *CkPIP1;2* | 289 | 30946.03 | 8.27 | 33.61 | 97.92 | 0.41 | plas |
| *CkPIP1;3* | 287 | 30882.94 | 8.97 | 32.04 | 97.28 | 0.383 | plas |
| *CkPIP1;4* | 287 | 30922.88 | 9.13 | 29.68 | 95.23 | 0.341 | plas |
| *CkPIP1;5* | 275 | 31133.81 | 9.92 | 29.8 | 109.89 | 0.585 | vacu |
| *CkPIP1;6* | 275 | 31115.78 | 9.92 | 29.8 | 111.31 | 0.592 | vacu |
| *CkPIP1;7* | 287 | 31012.86 | 8.97 | 23.78 | 92.82 | 0.249 | plas |
| *CkPIP2;1* | 287 | 30690.64 | 8.29 | 31.24 | 101.95 | 0.499 | plas |
| *CkPIP2;2* | 287 | 30782.58 | 7.65 | 35.85 | 97.25 | 0.41 | plas |
| *CkPIP2;3* | 285 | 30654.3 | 6.58 | 32 | 92.81 | 0.355 | plas |
| *CkPIP2;4* | 288 | 30712.69 | 8.81 | 32.86 | 102.05 | 0.457 | plas |
| *CkPIP2;5* | 187 | 19900.27 | 9.3 | 26.87 | 102.78 | 0.618 | plas |
| *CkPIP2;6* | 281 | 29833.79 | 9.07 | 31.12 | 100.36 | 0.494 | plas |

**Table. S3 CkPIP protein secondary structure information**

| Protein Name | Alpha helix（%） | Beta turn（%） | Random coil（%） | Extended strand（%） |
| --- | --- | --- | --- | --- |
| CkPIP1;1 | 30.80 | 1.73 | 52.60 | 14.88 |
| CkPIP1;2 | 31.49 | 1.38 | 50.52 | 16.61 |
| CkPIP1;3 | 32.40 | 1.39 | 51.22 | 14.98 |
| CkPIP1;4 | 33.10 | 1.39 | 50.52 | 14.98 |
| CkPIP1;5 | 36.73 | 1.45 | 43.64 | 18.18 |
| CkPIP1;6 | 35.64 | 3.64 | 43.27 | 17.45 |
| CkPIP1;7 | 30.66 | 1.05 | 51.92 | 16.38 |
| CkPIP2;1 | 33.10 | 2.09 | 48.43 | 16.38 |
| CkPIP2;2 | 32.75 | 1.05 | 49.13 | 17.07 |
| CkPIP2;3 | 31.93 | 1.75 | 49.47 | 16.84 |
| CkPIP2;4 | 31.25 | 1.39 | 51.04 | 16.32 |
| CkPIP2;5 | 32.62 | 2.14 | 44.39 | 20.86 |
| CkPIP2;6 | 34.16 | 1.07 | 49.47 | 15.30 |

**Table. S4 Interacting protein information**

| Protein ID | Predicted Functional Partners | Description |
| --- | --- | --- |
| GLYMA_20G170400 | 1NH56_S0YBN | Uncharacterized protein; Belongs to the MIP/aquaporin (TC 1.A.B) family. |
| GLYMA_02G165500 | 1JFY8_SOYBN | Glycerol-3-phosphate dehydrogenase; Belongs to the FAD-dependent glycerol-3-phosphate dehydrogenase family. |
| GLYMA_10G207700 | 1LCX0_SOYBN | Glycerol-3-phosphate dehydrogenase; Belongs to the FAD-dependent glycerol-3-phosphate dehydrogenase family. |
| GLYMA_20G183100 | 1NHI5_SOYBN | Glycerol-3-phosphate dehydrogenase; Belongs to the FAD-dependent glycerol-3-phosphate dehydrogenase family. |
| GLYMA_05G130700 | 1K345_SOYBN | AAA domain-containing protein. |
